# Effect of Deposition Parameters on Micromechanical Properties and Machining Performance of CrN Coating for Wet Finish Turning of Ti6Al4V Alloy

**DOI:** 10.3390/ma17174328

**Published:** 2024-08-31

**Authors:** Mohammad Shariful Islam Chowdhury, Bipasha Bose, Shahana Akter, Stephen Clarence Veldhuis

**Affiliations:** McMaster Manufacturing Research Institute (MMRI), Department of Mechanical Engineering, McMaster University, 1280 Main Street West, Hamilton, ON L8S 4L7, Canada; chowdhms@mcmaster.ca (M.S.I.C.); shahanaaktersheela@gmail.com (S.A.); veldhu@mcmaster.ca (S.C.V.)

**Keywords:** finish turning, CrN coating, Ti6Al4V alloy, built-up edge (BUE), flank wear

## Abstract

This study aims to optimize the performance of CrN coatings deposited on WC cutting tools for machining Ti6Al4V alloy, where the formation of built-up edge (BUE) is a prevalent and critical issue. In-house CrN coatings were developed using the PVD (Physical Vapor Deposition) process, with variations in deposition parameters including nitrogen gas pressure, bias voltage, and coating thickness. A comprehensive experimental approach encompassing deposition, characterization, and machining performance evaluation was employed to identify the optimal deposition conditions. The results indicated that CrN coatings deposited at a nitrogen gas pressure of 4 Pa, a bias voltage of −50 V, and a thickness of 1.81 µm exhibited superior performance, significantly reducing BUE formation and tool wear. These optimized coatings demonstrated enhanced properties, such as a higher elastic modulus and a lower coefficient of friction, which contributed to improved tool life and machining performance. Comparative studies with commercial CrN coatings revealed that the in-house developed coatings outperformed the commercial variants by approximately 65% in tool life, owing to their superior mechanical properties and reduced friction. This research highlights the potential of tailored CrN coatings for advanced machining applications and emphasizes the importance of optimizing deposition parameters to achieve high-performance tool coatings.

## 1. Introduction

PVD (Physical Vapor Deposition) coatings are commonly used in modern machining applications to address adhesion-related issues. For machining applications such as titanium machining, one of the main drawbacks is the tendency for the work material to stick to the tool body. Such intensive adhesion results in BUE (built-up edge) formation, and as BUE is an unstable structure it exhibits a temporary, avalanche-like behavior, ultimately leading to severe surface damage when it breaks down during cutting [1]. Since BUE formation is inevitable in machining, minimizing it is crucial for improving tool performance and achieving a smoother surface finish. The properties of a coating must be optimized to limit BUE formation, as these properties directly impact the performance of PVD coatings.

Numerous researchers have investigated the application of PVD-coated tools to improve performance in the machining of titanium alloys. However, widely used PVD coatings such as AlTiN, Al_2_O_3_, HfN, TiC, TiN, TiCN, TiN/TiC, TiN/TiC/TiN, and Al_2_O_3_/TiC often exhibit lower efficiency compared to uncoated tools [2,3,4]. This decline in efficiency is largely due to the formation of built-up edge (BUE), which leads to coating delamination under mechanical stress [5,6,7]. Some studies [8,9,10,11], however, have reported slight improvements in tool performance with the use of coatings such as TiN, cBN + TiAlN, TiN/TiCN/TiN, TiAlN/NbN, TiAlN, and AlCrN. Despite these findings, only a limited number of studies have concentrated on adjusting coating properties specifically to reduce BUE formation during the machining of titanium alloys.

The architecture of the coating, the deposition technique, and process variables like reactive gas pressure, bias voltage, deposition temperature, and table rotation speed influence the mechanical properties and performance of PVD coatings [12,13,14,15]. Adjusting these parameters affects ion bombardment energy, a crucial deposition variable that determines coating features such as residual stress, structure, and texture [16]. Consequently, the properties of a coating, including microstructure, hardness, elastic modulus, residual stress, and adhesion, can be tailored by altering deposition parameters [17,18,19,20] to suit a specific wear mode in machining applications [4]. For instance, Cheng-Hsun Hsu et al. [21] reported that by varying the bias parameters of the cathodic arc PVD process, the wear resistance of TiN/CrN coatings can be enhanced. Similarly, Bibeye Jahaziel Ronadson et al. [22] investigated the impact of varying nitrogen gas flow during reactive co-sputtering on the crystal structure and properties of CrNx-WS2 solid lubricant coatings. They found that the hardness, Young’s modulus, and friction coefficient of these coatings could be adjusted by altering the nitrogen content.

Previous studies have shown that CrN coatings are particularly effective in scenarios where BUE formation is common. Their chemical stability, excellent oxidation and corrosion resistance, and beneficial tribological properties, such as a low coefficient of friction and enhanced toughness, significantly reduce material adhesion. Achieving these properties requires a specific combination of hardness, elastic modulus, H/E ratio, and plasticity index in the coating. It was found that for addressing BUE formation, CrN coatings need an optimal combination of micro-mechanical properties—a low H/E ratio, high toughness, low roughness, and high plasticity index—achievable through modifications in deposition parameters [23,24].

In the current study, CrN coatings were deposited in-house on WC cutting tools using various deposition parameters. The best-performing coating was identified through a series of tests, characterization methods, and machining studies. This developed coating was then compared to an existing commercial CrN coating also deposited on WC cutting tools. The primary objective of this research was to develop a CrN PVD coating with optimal micromechanical and tribological properties for high-speed, wet finish turning of Ti6Al4V alloy.

## 2. Experimental Procedures

### 2.1. Design of Experiments

The study was divided into two parts. In the first part, CrN coating was deposited in-house on WC cutting tools while varying several deposition parameters (as explained in Section 2.1.1). The best-performing coating was identified through various tests, characterization methods, and machining studies. In the second part, the in-house best-performing coating was compared with commercially available options.

The performance of the CrN-coated WC cutting tools were evaluated by wet finish turning of Ti6Al4V alloy. In the following sections, all the experimental procedures implemented in both parts are discussed.

#### 2.1.1. Coating Deposition

In this study, all coatings were deposited on Kennametal CNGG432 and polished Sandvik Coromant SPGN120308 carbide uncoated insert. The Kennametal CNGG432 inserts were used for finish turning tests, while coating characterization was performed on the flat-polished Sandvik Coromant SPGN120308 inserts. Both types of inserts share the same cemented carbide grade and microstructural composition. The commercial CrN coating was provided by Oerlikon Balzers, Canada. Coating thicknesses for all coatings were measured using the Calotest method with a 25 mm steel ball.

The in-house CrN coatings were deposited at McMaster Manufacturing Research Institute PVD coating facility using an AIP-S20 PVD Coater (Kobelco, Kakogawa, Hyogo, Japan) with a 99.9% purity Cr target, 105 mm in diameter. Prior to deposition, the mirror-polished cemented carbide substrates were ultrasonically cleaned in acetone. After the pumping-down procedure (reaching a pressure of 10 × 10⁻^3^ Pa), in situ cleaning of the substrates was performed by argon ion etching with a substrate bias voltage of 400 V at 1.33 Pa pressure for 7.5 min. The Cr target operated in arc mode at 150 A, and the working table rotated at 5 rpm during the process. The substrate temperature was maintained at 500 °C during coating deposition, with N_2_ gas used as the process gas. The pressure of the process gas, bias voltage, and coating deposition time were varied to obtain CrN coatings with different combinations of deposition parameters. Table 1 details the variations in deposition parameters used to fine-tune the CrN coating.

#### 2.1.2. Coating Characterization

An Anton Paar-NHT3 Nanoindentation Tester (Anton Paar, Baden, Switzerland) was employed to determine the coatings’ hardness, elastic modulus, and yield stress. Forty nanoindentations were performed at room temperature using a Berkovich diamond indenter with a load of 50 mN to measure the hardness and elastic modulus.

The Anton Paar-RST3 Revetest^®^ Scratch Tester (Anton Paar, Baden, Switzerland) was used for micro-scratch tests to study the coatings’ failure behavior under progressive loading. These tests utilized a Rockwell diamond indenter with a 20 μm end radius, with loads progressively increasing from 0.5 N to 5 N over a scratch length of 0.5 mm. The scratching speed and loading rate were maintained at 0.78 mm/min and 7.020 N/min, respectively, with each test repeated three times per coating.

An Anton Paar-TRB3 Pin-on-Disk tribometer (Anton Paar, Baden, Switzerland) was used to measure the coefficient of friction of the coatings. Tests were performed with a 6 mm alumina ball under a normal load of 20 N, covering a total distance of 250 m, with an amplitude of 4 mm, and a frequency of 8 Hz.

The toughness of the coatings was measured using a ZwickRoell Durascan 50 G5 (ZwickRoell, Ulm, Germany) with an applied load of 5 kg. Toughness values were calculated based on the load and the total crack lengths, measured from the crack tip to the corner of each indent.

A Bruker D8-Discover equipped with a 2D detector VAATEC-500 (Bruker, Billerica, MA, USA) was used to investigate the phase composition and crystal structure of the coatings. The radiation source was Cu-Kα (1.540 Å), with operating conditions of 40 kV and 40 mA. LEPTOS 7.8 software was used for residual stress calculation, and DIFFRAC.EVA V6.0 for phase identification. The grazing incidence diffraction method was employed for phase identification and residual stress measurement, performing a comprehensive scan over an angular range of 10° to 90° with an incidence angle of 2°.

#### 2.1.3. Machining Studies

**Figure 1:**
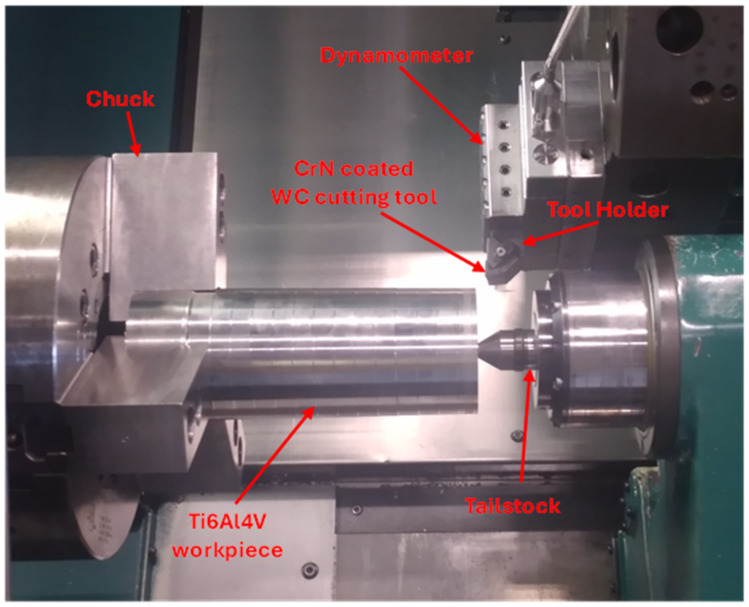
Experimental setup for the wet finish turning experiments.

To assess machining performance, progressive tool wear studies were conducted on a cylindrical ASTM B265 Grade 5 Ti6Al4V alloy workpiece with an initial diameter of 255 mm, using an MCLN-5° Kennametal Kenloc™ tool holder and CNGG432 carbide grade (WC, 6% Co) K313 inserts from Kennametal. XTREME CUT 290 semi-synthetic cutting fluid was used during the test. Table 2 outlines the cutting parameters used, which were based on industry recommendations. All tests were repeated three times for each coating to ensure repeatability. Figure 1 shows the experimental setup for the turning operation. Flank wear measurements (VB_max_) were made using a Keyence optical microscope (model VHX-5000 Series, KEYENCE America, Elmwood Park, NJ, USA) during the tests, with a tool life measurement scatter of approximately 10%.

Volumetric wear measurements were taken after approximately every 600 m of machining length using an Alicona Infinite Focus G5 3D surface measurement system (Alicona Manufacturing Inc., Bartlett, IL, USA). Surface roughness measurements of the coatings were also taken using this instrument.

SEM images were captured with a Vega 3-TESCAN scanning electron microscope (SEM) (Tescan, Brno Kohoutovice, Czech Republic). ImageJ 1.54g software was used to calculate the area fraction of macroparticle density and the area fraction of porosity on the coated surfaces.

Cutting forces during machining were measured using a Kistler Type 9129AA 3-component piezoelectric dynamometer, with a sampling rate of 10 kHz. Data processing was conducted using LabVIEW 2014.

## 3. Results and Discussions

### 3.1. In-House Coating Development and Characterization

This section provides a summary of all experimental results pertaining to the in-house developed coating.

#### 3.1.1. Tool Wear Performance Studies

The tuning of the deposition parameters (nitrogen gas pressure, bias voltage, and coating thickness) for the MMRI CrN coating was performed sequentially based on tool performance. Initially, the nitrogen gas pressure was optimized while keeping the bias voltage and coating thickness constant. Next, the bias voltage was fine-tuned with the coating thickness constant and the nitrogen gas pressure set to its optimal value. Finally, the coating thickness was adjusted, maintaining the optimal values for both nitrogen gas pressure and bias voltage.

##### Tuning of Nitrogen Gas Pressure

**Figure 2:**
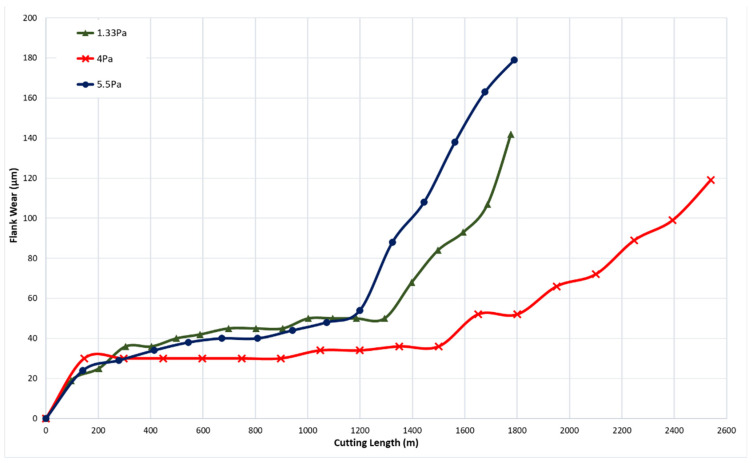
Flank wear versus cutting length data during finish turning of Ti6Al4V alloy using CrN coatings applied under different nitrogen gas pressures.

Three CrN coatings were deposited under nitrogen gas pressures of 1.33 Pa, 4 Pa, and 5.5 Pa. Throughout the depositions, the bias voltage was consistently set at −50 V, and the coating thickness was maintained at approximately 1.8 µm. Figure 2 presents the tool wear performance for the coatings deposited at different nitrogen gas pressures. The coating with a nitrogen gas pressure of 4 Pa showed the lowest flank wear intensity, followed by those at 1.33 Pa and 5.5 Pa.

**Figure 3:**
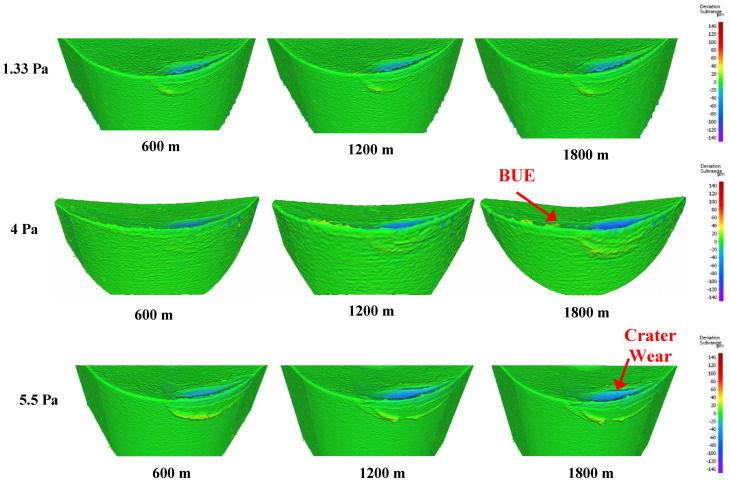
Volumetric progressive wear measurement of CrN coated tools applied under different nitrogen gas pressures, showing the development of built-up layers and crater wear during machining.

**Figure 4:**
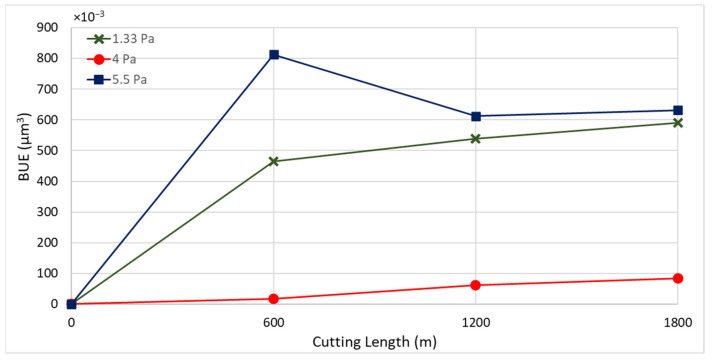
Progression of built-up volume versus length of cut for CrN coated tools applied under different nitrogen gas pressures, considering the peaks above the original tool’s reference surface.

**Figure 5:**
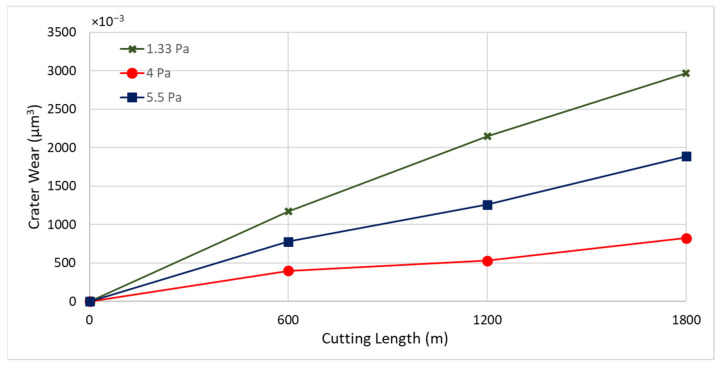
Progression of crater wear volume versus length of cut for CrN coated tools applied under different nitrogen gas pressures, considering the peaks below the original tool’s reference surface.

Figure 3 displays the progression of tool wear and the 3D difference measurements of worn tools after every 600 m of machining length for coatings deposited at different nitrogen gas pressures. The 3D images clearly show that tool wear was influenced by built-up edge (BUE) formation and crater wear. Figure 4 and Figure 5 illustrate the variation in BUE formation and crater wear for the different coatings. As shown in Figure 3, Figure 4 and Figure 5, the CrN coating deposited at 4 Pa nitrogen gas pressure exhibited the least BUE formation and crater wear progression. Reduced BUE formation and crater wear during machining minimize the likelihood of tool chipping. The lower BUE formation, along with delayed and reduced crater wear intensity, resulted in stable, uniform, and minimal tool wear for the CrN coating deposited at 4 Pa nitrogen gas pressure. Therefore, 4 Pa is determined to be the optimal nitrogen gas pressure for CrN coating deposition.

##### Tuning of Bias Voltage

**Figure 6:**
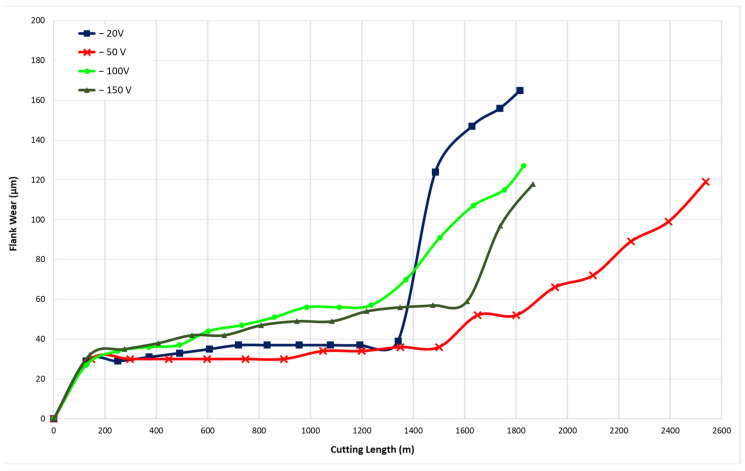
Flank wear versus cutting length data during finish turning of Ti6Al4V alloy using CrN coatings applied under different bias voltages.

To identify the optimal bias voltage for CrN coating deposition, four CrN coatings were applied at bias voltages of −20 V, −50 V, −100 V, and −150 V. All coatings were deposited at a nitrogen gas pressure of 4 Pa, which had previously been determined to be optimal, and a coating thickness of approximately 1.8 µm. Figure 6 illustrates the tool wear performance of the coatings deposited at these varying bias voltages. The CrN coating deposited at a bias voltage of −50 V demonstrated the best tool performance.

**Figure 7:**
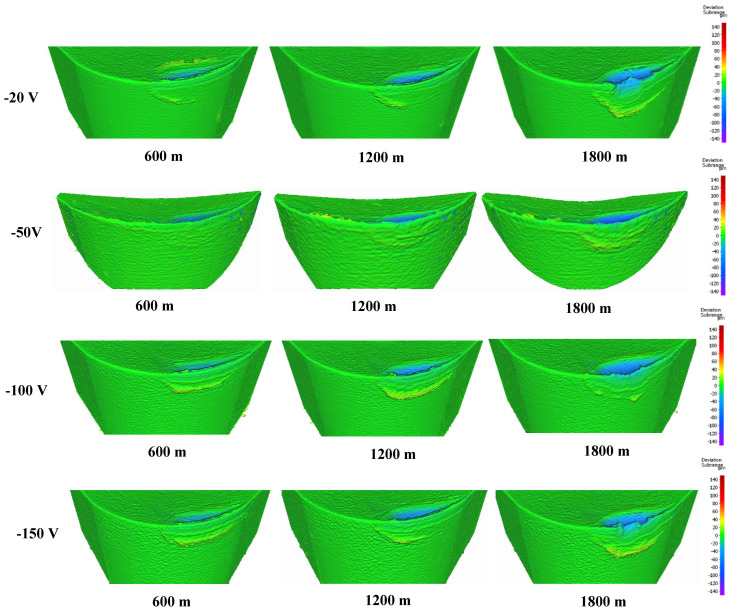
Volumetric progressive wear measurement of CrN coated tools applied under varying bias voltages, showing the development of built-up layers and crater wear during machining.

**Figure 8:**
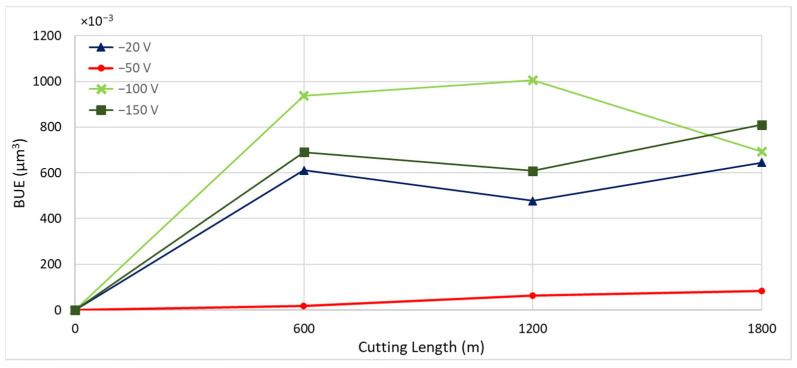
Progression of built-up volume versus length of cut for CrN coated tools applied under varying bias voltages, considering the peaks above the original tool’s reference surface.

**Figure 9:**
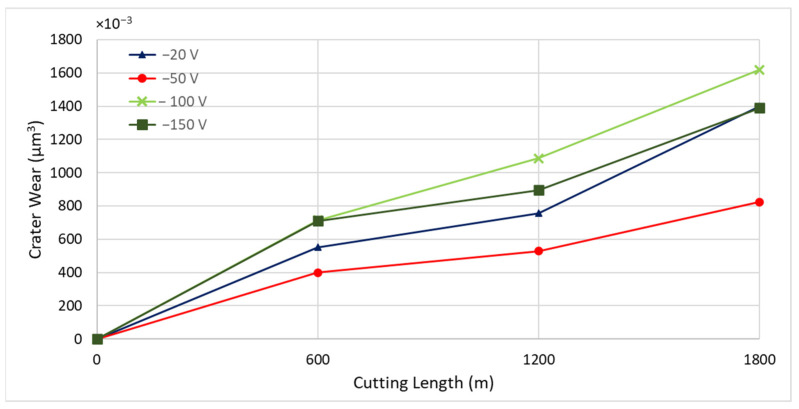
Progression of crater wear volume versus length of cut for CrN coated tools applied under varying bias voltages, considering the peaks below the original tool’s reference surface.

Figure 7 presents the tool wear progression and the 3D volume measurements of the worn tool after approximately every 600 m of machining length for coatings deposited at different bias voltages. Figure 8 and Figure 9 show the formation of BUE and crater wear as a function of cutting length for the various coatings. The CrN coating deposited at a bias voltage of −50 V exhibited the least BUE formation and crater wear progression during cutting. Reduced BUE formation and delayed crater wear led to a steady increase in tool wear, preventing rapid tool failure. This indicates that a bias voltage of −50 V is optimal for CrN coatings deposited using the cathodic arc evaporation method with an AIP-S20 PVD deposition system.

##### Tuning of Coating Thickness

**Figure 10:**
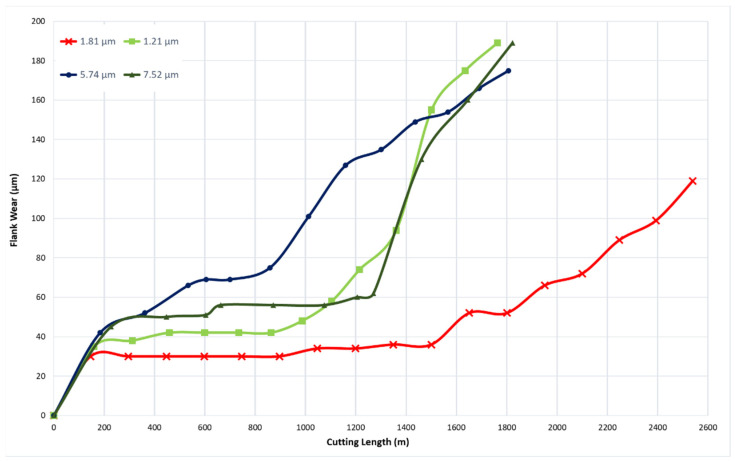
Flank wear versus cutting length data during finish turning of Ti6Al4V alloy using CrN coatings of varying thicknesses.

To determine the optimal coating thickness for CrN coatings to enhance machining performance, four coatings were deposited with thicknesses of 1.21 µm, 1.81 µm, 5.74 µm, and 7.52 µm. All depositions were conducted at a nitrogen gas pressure of 4 Pa and a bias voltage of −50 V, which were previously identified as the optimal conditions. Figure 10 illustrates the tool wear performance for the coatings with different thicknesses. The coating with a thickness of 1.81 µm demonstrated the best tool performance.

**Figure 11:**
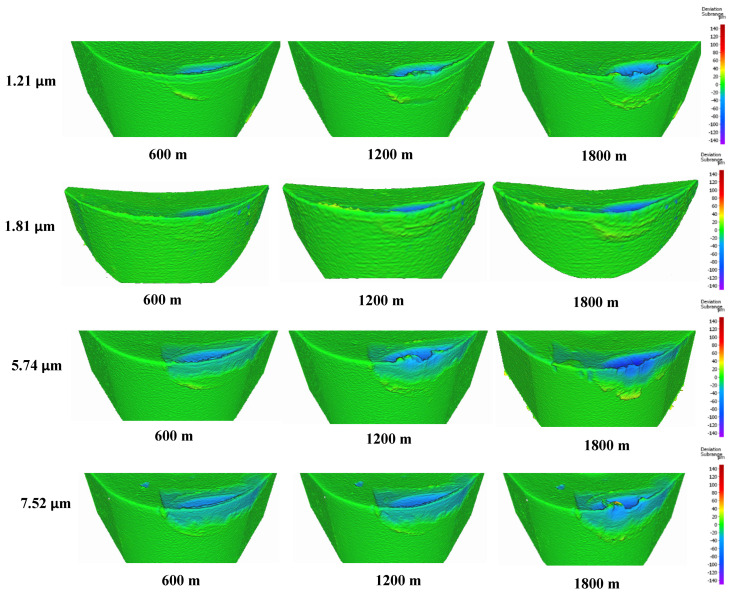
Volumetric progressive wear measurement of CrN coated tools of varying thicknesses, showing the development of built-up layers and crater wear during machining.

**Figure 12:**
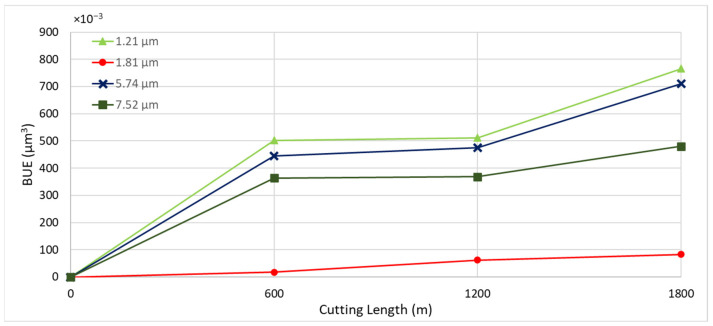
Progression of built-up volume versus length of cut for CrN coated tools of varying thicknesses, considering the peaks above the original tool’s reference surface.

**Figure 13:**
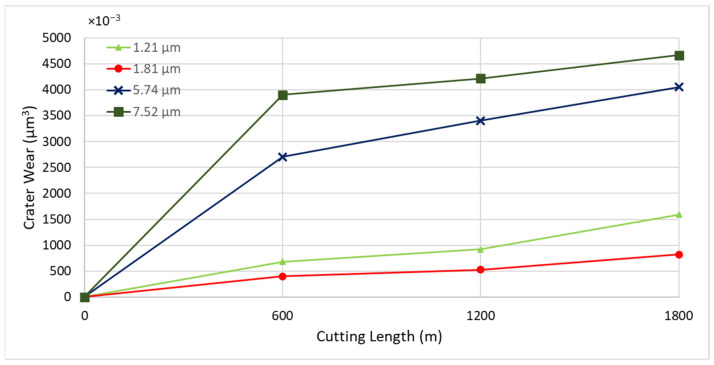
Progression of crater wear volume versus length of cut for CrN coated tools applied under varying thicknesses, considering the peaks below the original tool’s reference surface.

Figure 11 shows the progression of tool wear and the 3D volume measurement of the cutting tool after every 600 m of cutting length for the deposited coatings with varying thicknesses. Figure 12 and Figure 13 illustrate the formation of BUE and crater wear relative to cutting length for the different coatings. As shown, the CrN coating with a thickness of 1.81 µm exhibited the least BUE formation and crater wear progression during cutting. Significant delamination occurred in the CrN coatings with thicknesses of 5.74 µm and 7.52 µm. Therefore, a coating thickness of approximately 1.81 µm is optimal for this specific application.

Based on machining performance, the CrN coating deposited with a nitrogen gas pressure of 4 Pa, a bias voltage of −50 V, and a coating thickness of 1.81 μm yielded the best tool performance. This combination of deposition parameters resulted in the minimal formation of BUE on the coated tool and reduced crater wear progression.

#### 3.1.2. Coating Characterization

**Figure 14:**
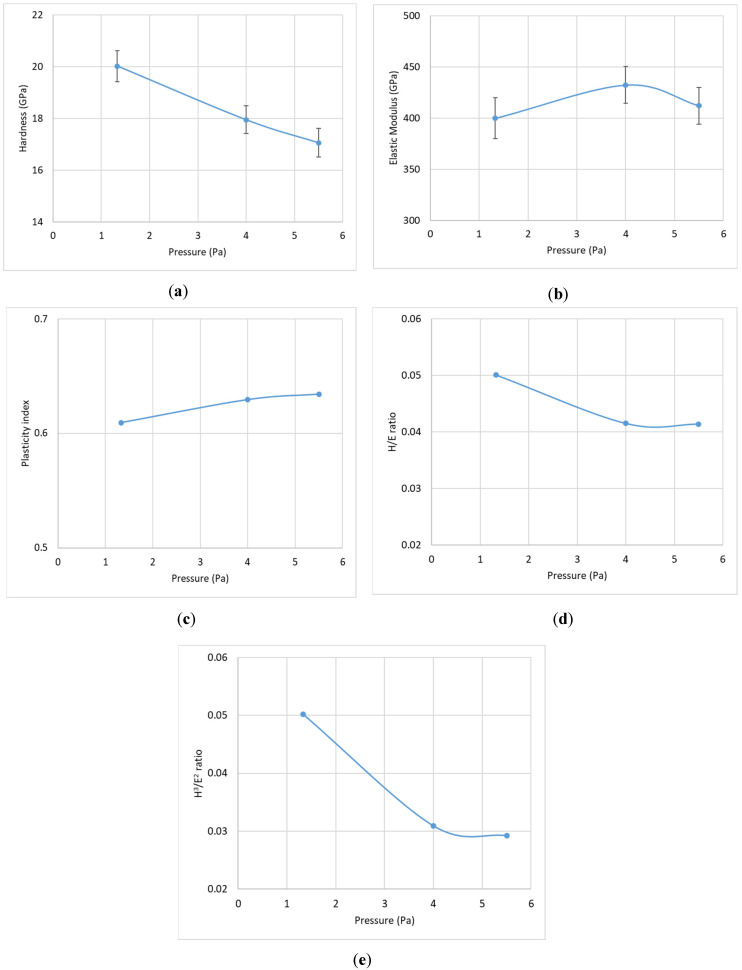
Variation in micromechanical properties of CrN coatings applied under different nitrogen gas pressures: (**a**) hardness, (**b**) elastic modulus, (**c**) plasticity index, (**d**) H/E ratio, and (**e**) H^3^/E^2^ ratio.

**Figure 15:**
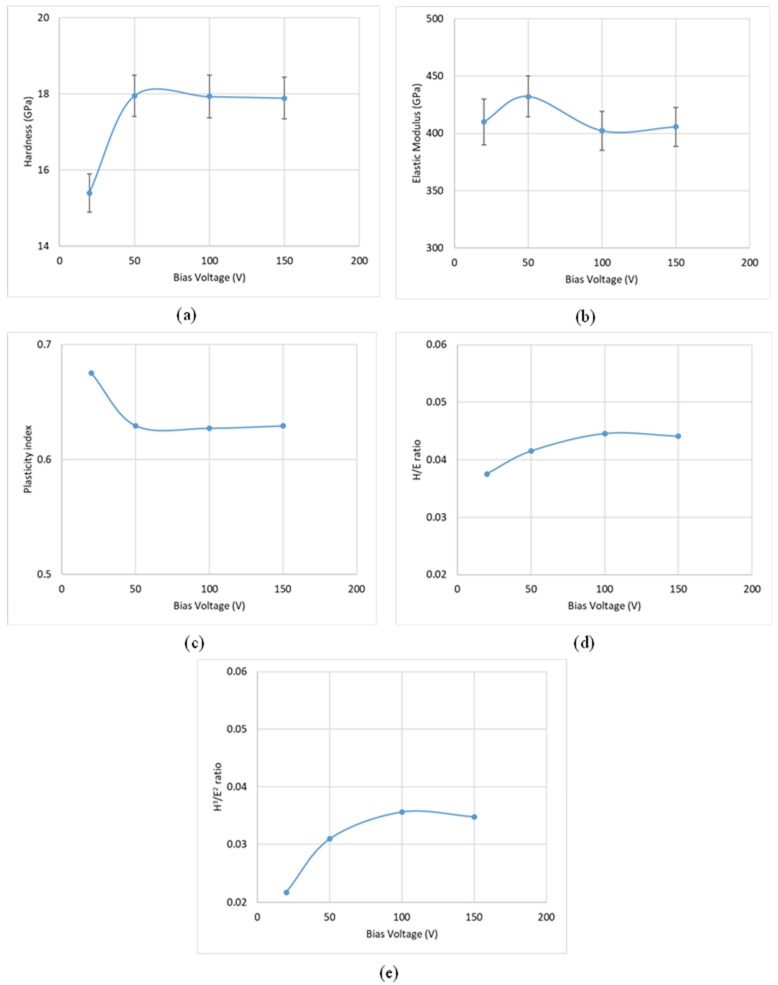
Variation in micromechanical properties of CrN coatings applied under different bias voltages: (**a**) hardness, (**b**) elastic modulus, (**c**) plasticity index, (**d**) H/E ratio, and (**e**) H^3^/E^2^ ratio.

**Figure 16:**
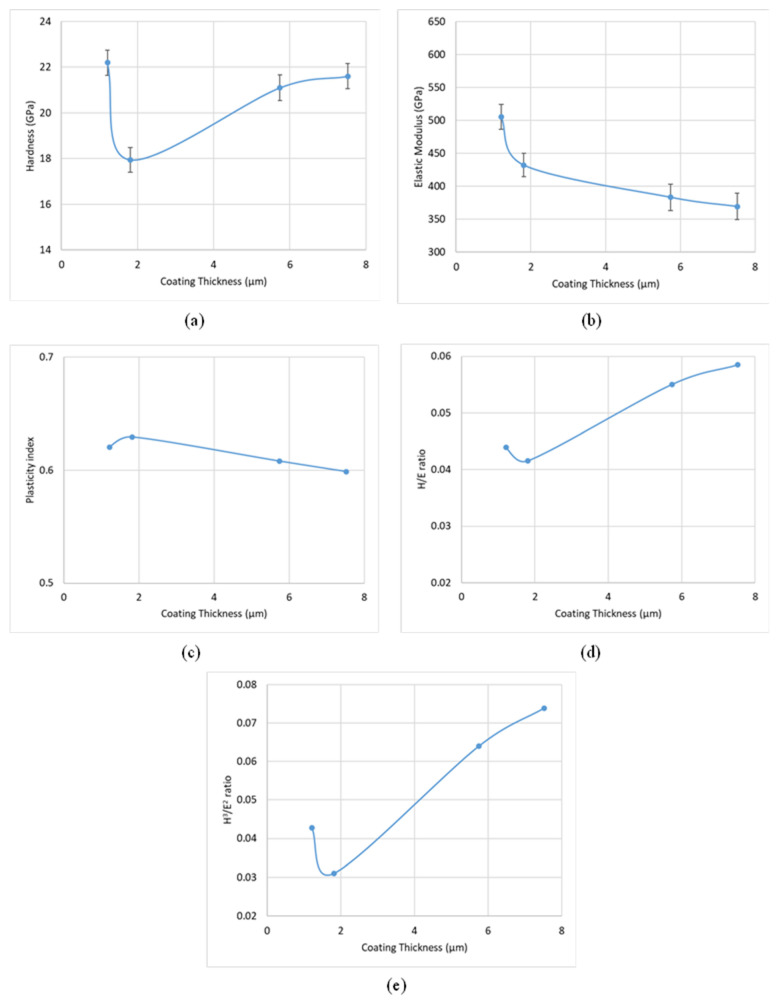
Variation in micromechanical properties of CrN coatings of varying thicknesses: (**a**) hardness, (**b**) elastic modulus, (**c**) plasticity index, (**d**) H/E ratio, and (**e**) H^3^/E^2^ ratio.

As detailed earlier, the CrN coating deposited at a nitrogen gas pressure of 4 Pa, a bias voltage of −50 V, and a coating thickness of 1.81 μm exhibited the best tool performance. To further investigate the properties and failure behavior of these coatings under load, micromechanical studies were conducted. Figure 14, Figure 15 and Figure 16 display the variations in hardness, elastic modulus, plasticity index, H/E ratio, and H^3^/E^2^ ratio for coatings deposited with different nitrogen gas pressures, bias voltages, and coating thicknesses.

Coatings deposited at 4 Pa and −50 V, which provided optimal tool performance, showed higher elastic modulus values. Previous studies [25] have indicated that coatings with slightly lower hardness but higher elastic modulus tend to perform better during Ti6Al4V machining. Research [26,27] has also demonstrated that wear resistance improves with increased elastic modulus. A similar trend is observed with variations in coating thickness. Specifically, as shown in Figure 16a,b, a thickness of 1.81 μm results in the lowest hardness value while maintaining a relatively high elastic modulus.

The H/E ratio reflects the coating’s ability to remain elastic during mechanical contact, while the H^3^/E^2^ ratio indicates resistance to plastic deformation [28,29]. The plasticity index, which measures the ratio of plastic to total work done during nanoindentation [30], assesses the coating’s energy dissipation capacity. Figure 14, Figure 15 and Figure 16c–e illustrate that for the best-performing coating with 4 Pa deposition pressure, –50 V bias voltage, and 1.81 μm thickness, the values for plasticity index, H/E, and H^3^/E^2^ ratios fall between the extremes, suggesting that an optimized balance between hardness and elastic modulus is more beneficial for tool performance than maximizing either parameter. The literature [31] supports the notion that an optimized combination of hardness and elastic modulus is crucial for extended tool life, especially in high-load applications such as titanium machining.

### 3.2. Comaprison of In-House Developed Coating with Commercial Coating

In the previous section, the CrN coating deposited at 4 Pa pressure, with a −50 V bias voltage, and a thickness of 1.81 µm, was identified as the top-performing among all the in-house developed coatings. In this section, this best-performing in-house coating was compared to a commercially available CrN coating.

#### 3.2.1. Tool Wear Performance Studies

**Figure 17:**
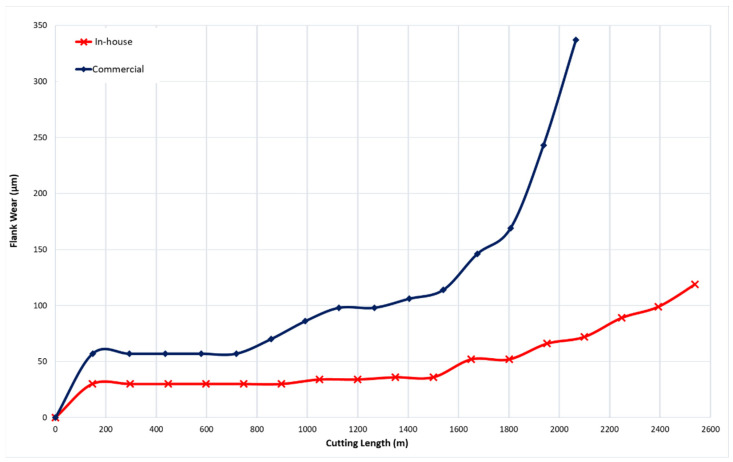
Flank wear versus cutting length data during finish turning of Ti6Al4V alloy using in-house and commercial CrN coatings.

The performance of the in-house developed CrN coating was compared with a commercial CrN coating for finish turning operations. Figure 17 shows the tool wear progression as a function of cutting length for both the in-house and commercial CrN-coated tools. The commercial CrN coating displayed the highest wear intensity, while the in-house CrN coating provided a tool life improvement of approximately 65% over the commercial coating.

**Figure 18:**
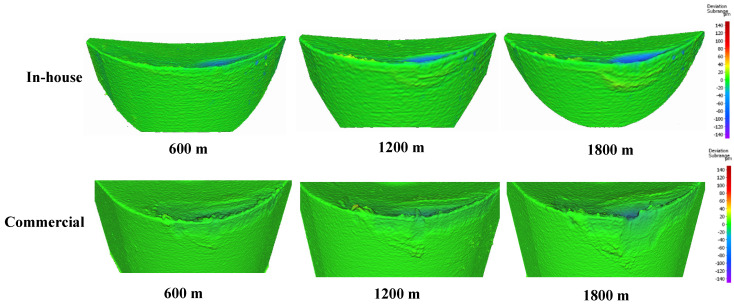
Volumetric progressive wear measurement of in-house and commercial CrN coatings, showing the development of built-up layers and crater wear during machining.

Progressive tool wear studies were performed every 600 m of cutting length using 3D imaging with the Alicona system. Figure 18 depicts the tool wear progression for both the in-house and commercial CrN-coated tools. Two primary wear phenomena were observed: pronounced adhesive interaction at the tool–chip interface and crater wear. The 3D images revealed notable build-up edge (BUE) formation and crater wear propagation in both coating types as cutting continued.

**Figure 19:**
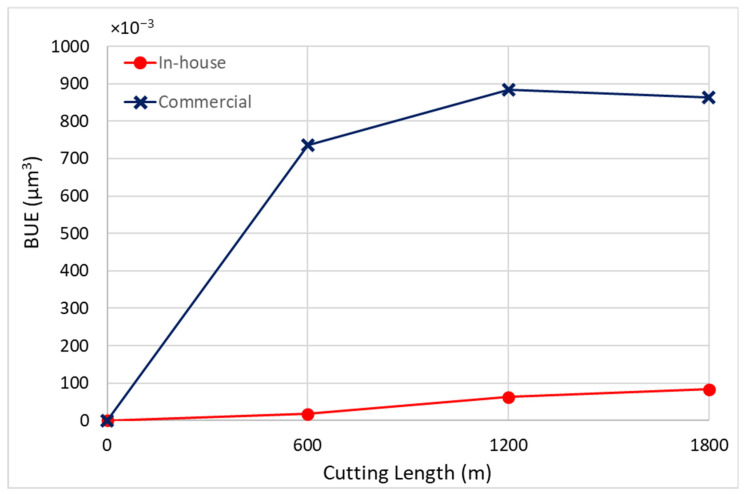
Progression of built-up volume versus length of cut for in-house and commercial CrN coatings, considering the peaks above the original tool’s reference surface.

**Figure 20:**
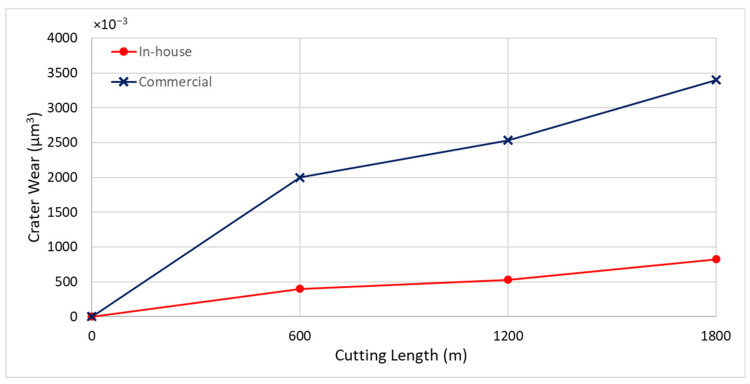
Progression of crater wear volume versus length of cut for in-house and commercial CrN coatings, considering the peaks below the original tool’s reference surface.

Figure 19 and Figure 20 illustrate the BUE formation and crater wear relative to cutting length for the coated tools. The commercial CrN coating exhibited significant BUE formation compared to the in-house CrN coating.

**Figure 21:**
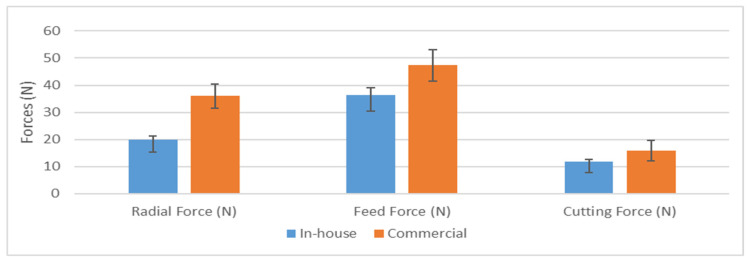
Variation in cutting forces for in-house and commercial CrN coatings.

The reduced BUE formation observed with the in-house CrN coating diminished the risk of tool edge chipping and contributed to decreased crater wear intensity and a delay in its progression. Consequently, the in-house CrN coating led to a more consistent increase in tool wear, effectively preventing rapid tool failure. This is corroborated by cutting force measurements as shown in Figure 21. The in-house coated tool exhibits lower cutting forces in all directions compared to the commercial coated tool. This reduction can be attributed to the lower BUE formation and reduced friction at the tool–chip interface.

#### 3.2.2. Coating Characterization

Thorough evaluations were performed to examine the mechanical and structural properties of both in-house and commercial CrN coatings. X-ray diffraction (XRD) analysis revealed that all coatings exhibited Face-Centered Cubic (FCC) structures, with prominent peaks corresponding to the crystallographic planes (111), (200), (220), and (311). Among these, the (200) peak showed the highest intensity in the XRD spectra, suggesting that the (200) plane was the preferred orientation for all the CrN coatings.

**Figure 22:**
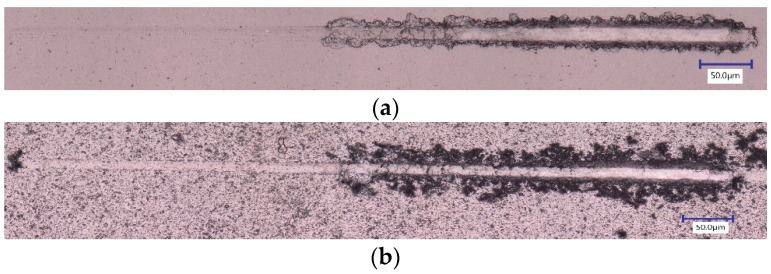
Optical images of the scratch track for CrN coatings: (**a**) In-house, (**b**) Commercial.

Micromechanical studies comparing the two coatings are summarized in Table 3. Coatings deposited in-house at 4 Pa and −50 V, which provided optimal tool performance, exhibited a higher elastic modulus value and a slightly lower but comparable hardness value. As mentioned in the previous section, coatings with a higher elastic modulus, resulting in a slightly lower H/E ratio, tend to perform better during Ti6Al4V machining. The lower H^3^/E^2^ ratio, along with higher toughness values and plasticity index, indicates a tougher coating. This means the coating may yield earlier than the commercial one, but it will be less brittle, leading to better adhesion with the substrate and a longer lifespan. This is further confirmed by scratch tests, which show that while the in-house coating begins to fail at a similar load, the commercial coating fails in a brittle manner compared to the in-house coating (Figure 22). Additionally, the higher compressive residual stress values for the in-house coating also indicate better adhesion, as compressive residual stress can improve the coating’s adherence to the substrate, reducing the likelihood of delamination or peeling. It can also enhance the coating’s resistance to fatigue and crack propagation, contributing to its longevity and reliability, which is further supported by the coating’s performance in the machining studies.

**Figure 23:**
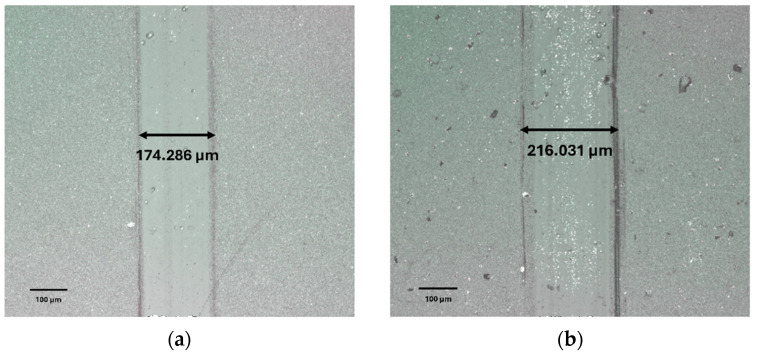
Wear track created during tribometer tests for the CrN coatings: (**a**) In-house, (**b**) Commercial.

The tribometer tests reveal that the in-house coating exhibits a lower coefficient of friction compared to the commercial coating (see Table 3). This indicates that the in-house coating has higher lubricity which is beneficial as this also reduces friction at the tool–chip interface during machining. This compliments the cutting force measurement data as shown earlier (Figure 21). The thinner wear track (Figure 23) is also indicative of better wear resistance ability of the in-house coating.

**Figure 24:**
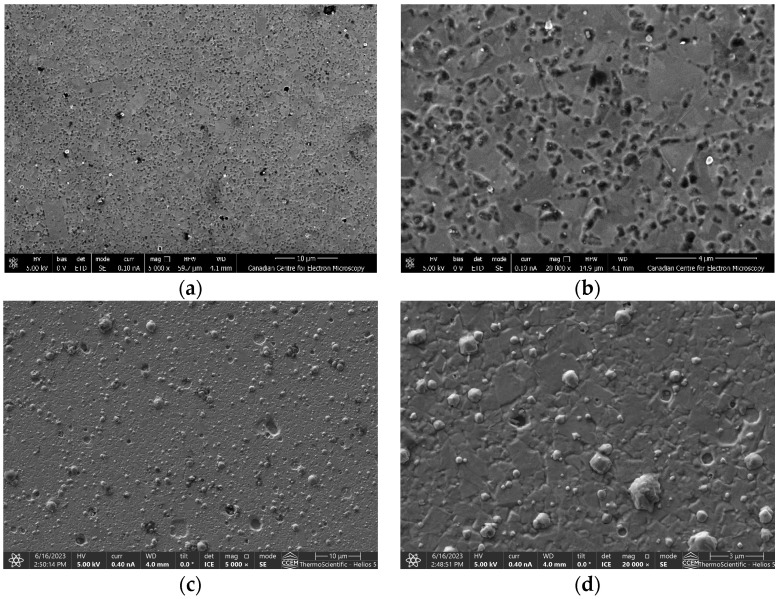
SEM images of CrN coatings’ surface morphology at different magnifications: In-house coatings at lower (**a**) and higher (**b**) magnification; Commercial coatings at lower (**c**) and higher (**d**) magnification.

Figure 24 shows the SEM images of the surface morphology of the in-house CrN coating and the commercial coating. The macroparticle droplets were higher for the commercial coating compared to the in-house coating, which contributed to the higher roughness value observed for the commercial coating (Table 3). Microparticle droplets are generally considered undesirable in PVD coatings due to their potential to impact surface quality, adhesion, and uniformity. For high-performance coatings, minimizing microparticle droplets is usually crucial to ensure the desired properties and reliability of the coating.

**Figure 25:**
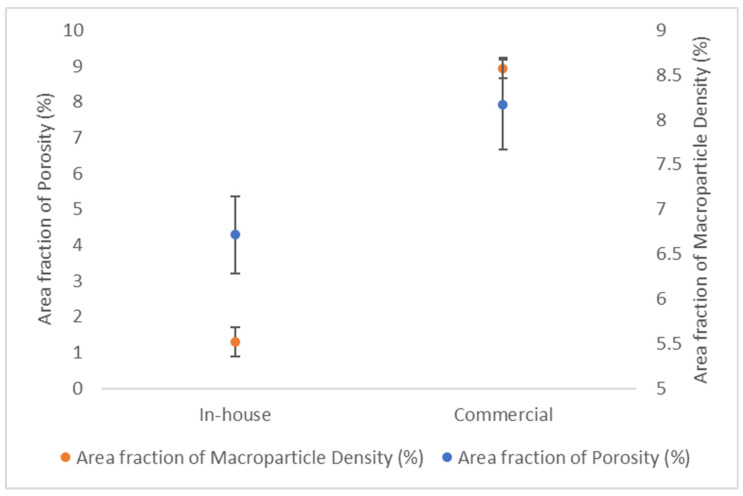
Area fraction of porosity and area fraction of macroparticle density for the commercial and in-house CrN coatings.

Porosity in PVD coatings can have significant impacts on the coating’s performance and durability and thus should be minimized. From Figure 25, it can be seen that both the area fraction of macroparticle density and the area fraction of porosity (determined using the ImageJ software) were higher for the commercial coating than for the in-house deposited coating.

## 4. Conclusions

The study has underscored the critical role of optimizing deposition parameters to enhance the performance of CrN coatings for machining Ti6Al4V alloy. The identified optimal conditions—4 Pa nitrogen gas pressure, −50 V bias voltage, and a coating thickness of 1.81 µm—resulted in significant improvements in tool performance, particularly in reducing built-up edge (BUE) formation and tool wear.

The CrN coating produced under these optimal conditions demonstrated remarkable advancements in tool performance. Compared to coatings deposited under non-optimal parameters, the optimized CrN coating exhibited minimal BUE formation and crater wear during machining. This enhanced performance is attributed to the superior mechanical and tribological properties of the coating, including increased hardness, elastic modulus, and plasticity index.

Notably, the in-house developed CrN coating showed a 65% improvement in tool life compared to a commercially available CrN coating. This superior performance is due to the optimized micro-mechanical properties and reduced friction at the tool–chip interface, which resulted in lower cutting forces and better wear resistance. Detailed characterization revealed that the optimized coating had a higher elastic modulus and a lower H/E ratio, contributing to better tool performance. Coatings with a lower H/E ratio and higher plasticity index exhibited improved resistance to BUE formation and crater wear. The enhanced hardness and toughness of the optimized coating also played a crucial role in extending tool life.

Micromechanical studies indicated that coatings deposited under optimal conditions had a balanced combination of hardness and elastic modulus, which is critical for high-performance machining. The optimal coating had a favorable H^3^/E^2^ ratio and plasticity index, suggesting an effective balance between hardness and elasticity that enhances its performance during machining.

Tribological tests further confirmed that the optimized CrN coating had a lower coefficient of friction compared to the commercial coating, leading to reduced wear and improved performance during cutting operations. Scanning Electron Microscopy (SEM) analysis showed fewer macroparticles and lower porosity in the in-house coating, contributing to improved surface quality, adhesion, and overall durability.

This research contributes valuable insights into the optimization of CrN coatings for high-speed machining, emphasizing the importance of tailoring deposition parameters to achieve desired coating properties. The improved performance of the in-house developed coating highlights the potential for customized coatings to significantly enhance tool life and machining efficiency in challenging applications.

## Figures and Tables

**Table 1:** Variation of deposition parameters.

N_2_ Gas Pressure (Pa)	Bias Voltage (V)	Coating Thickness (μm)
1.33, 4, 5.5	−20, −50, −100, −150	1.21, 1.81, 5.74, 7.52

**Table 2:** Cutting data for the experiments performed.

Machining Operation	Speed, m/min	Feed, mm/rev	Depth of Cut, mm	Coolant Condition
Finish Turning	150	0.1225	0.25	Flood

**Table 3:** Micromechanical properties of in-house and commercial CrN coatings.

Nomenclature	CrN Coating	
	In-House	Commercial
Thickness, µm	1.81 ± 0.1	2.01 ± 0.2
Hardness, GPa	17.95 ± 0.54	18.7 ± 3.6
Elastic Modulus, GPa	432.27 ± 18	346.86 ± 85.3
H/E Ratio	0.042	0.054
H^3^/E^2^ ratio	0.031	0.055
Plasticity Index	0.63	0.53
Mean coefficients of friction, μ	0.15 ± 0.04	0.43 ± 0.03
Modified Palmqvist Toughness, N/μm	1.23 ± 0.05	0.86 ± 0.03
Roughness Sa, µm	0.12 ± 0.15	0.22 ± 0.38
Residual Stress, MPa	−761.5 ± 153.7	−538.9 ± 117.0

## Data Availability

All data are presented in the current manuscript. For further query, communicate with the corresponding author.
